# Immunological Interactive Effects between Pollen Grains and Their Cytoplasmic Granules on Brown Norway Rats

**DOI:** 10.1097/WOX.0b013e3181b71bee

**Published:** 2009-09-15

**Authors:** Oussama R Abou Chakra, Sutra Jean-Pierre, Françoise Rogerieux, Gabriel Peltre, Hélène Sénéchal, Ghislaine Lacroix

**Affiliations:** 1 LECA-ESPCI, Paris, France; 2 INERIS, Department of Experimental Toxicology, Verneuil-en-Halatte, France; 3 INSERM, Paris, France

**Keywords:** immunologic interactive effects, timothy grass pollen, pollen cytoplasmic granules, allergy, inflammation

## Abstract

**Background:**

Grass pollen is one of the most important aeroallergen vectors in Europe. Under some meteorological factors, pollen grains can release pollen cytoplasmic granules (PCGs). PCGs induce allergic responses. Several studies have shown that during a period of thunderstorms the number of patients with asthma increases because of higher airborne concentrations of PCGs.

**Objective:**

The aims of the study were to assess the allergenicity of interactive effects between pollen and PCGs and to compare it with allergenicity of Timothy grass pollen and PCGs in Brown Norway rats.

**Methods:**

Rats were sensitized (day 0) and challenged (day 21) with pollen grains and/or PCGs. Four groups were studied: pollen-pollen (PP), PCGs-PCGs (GG), pollen-PCGs (PG), and PCGs-pollen (GP). Blood samples, bronchoalveolar lavage fluid, and bronchial lymph node were collected at day 25. IgE and IgG1 levels in sera were assessed by enzyme-linked immunosorbent assay. Alveolar cells, protein, and cytokine concentrations were quantified in bronchoalveolar lavage fluid. T-cell proliferation, in response to pollen or granules, was performed by lymph node assay.

**Results:**

Interactive effects between pollen and PCGs increased IgE and IgG1 levels when compared with those of the negative control. These increases were lower than those of the PP group but similar to the levels obtained by the GG group. Whatever was used in the sensitization and/or challenge phase, PCGs increased lymphocyte and Rantes levels compared with those of the pollen group. The interactive effects increased IL-1*α *and IL-1*β *compared with those of the PP and GG groups.

**Conclusions:**

Immunologic interactive effects have been shown between pollen and PCGs. For humoral and cellular allergic responses, interactive effects between the 2 aeroallergenic sources used in this study seem to be influenced mainly by PCGs.

## Introduction

Grass pollen is one of the most important aeroallergen vectors in the world, inducing respiratory allergic diseases such as asthma and hay fever. These diseases are currently a major public health problem worldwide, in particular in industrialized countries. In recent decades, epidemiological studies have shown that the prevalence of allergic diseases has dramatically increased, especially in children and adolescents and those living in urban areas [1].

These atopic diseases are complex inflammatory disorders influenced by both genetic and environmental factors, among which the atmospheric dispersion of pollen grains seems to take an important role. However, although the symptoms associated with these affections coincide with the pollination season, it is now well established that a simple and direct relationship does not exist between these 2 phenomena and that several other factors must also be taken into account [2].

In parallel, some studies have shown that, under specific meteorological factors such as thunderstorms or light rainfall and also contamination with airborne pollutants, pollen grains can release into the atmosphere several hundred small particles called pollen cytoplasmic granules (PCGs) [3,4]. The likelihood that whole grass pollen grains (15-60 *μ*m) will reach the deeper human airways is low; however, PCGs (< 5 *μ*m) might penetrate the lower respiratory tract and so induce symptoms of bronchial asthma and/or hay fever [5,6]. In fact, several studies noted an exacerbation of the number of patients with asthma after thunderstorms because of higher airborne concentrations of PCGs after such meteorological episodes [7,8].

Experimental studies have shown that PCGs can induce allergic reactions in pollen and PCGs sensitized animals [9,10]. Furthermore, PCGs can elicit a positive skin prick test for patients with allergies and may cause a bronchial obstruction, during periods of thunderstorms for those suffering from asthma [4].

Although most major allergens, Phl p 4 and Phl p 5, were present in the pollen grains (in the cytoplasmic matrix and in the membranes) and in their PCGs,[11,12] other allergens, such as Phl p 6 and Phl p 13, were associated only with PCGs [13,14]. For these reasons, the goal of this study was to evaluate, with a rat allergy model, the allergenic potential of interactive effects between pollen and PCGs and to compare them with the allergenicity of whole timothy grass pollen and PCGs individually. Humoral, cellular, and inflammatory responses have been assessed after intratracheally instillation of PCGs and/or whole pollen in Brown Norway rats.

## Methods

### (1) Animals

Six-week-old male Brown Norway (BN) rats were obtained from Charles River Laboratories (France). Animals were housed in the INERIS animal-care unit, a facility accredited by the Departmental Direction of Veterinary Services. They had free access to conventional laboratory feed and water. They were handled in accordance with French State Council guidelines for the care and use of laboratory animals (Decree 87-849, October 19, 1987) and experiments were approved by the Institutional Animal Care and Use Comity at the INERIS.

### (2) Pollen and PCGs

#### (a) Pollen grains

Pollen grains from Timothy grass (*Phleum pratense*) were collected on Allerbio AB site (Varennes-en-Argonne, France) and immediately sent to our laboratory after the harvest. This pollen had not undergone post-harvest conservative treatment and was kept in the best conditions (+4°C).

#### (b) PCGs

PCGs (0.6-5 *μ*m) were isolated from *Phleum pratense *pollen by osmotic shock in pure water after filtration, centrifugation, and 2 washes in distilled water. PCGs were resuspended in saline solution. They were counted with a particle counter (Z3 multisizer, Beckman Coulter).

After 2 washes of PCGs issued from 100 mg of pollen grains, the water-soluble protein concentration went down from 20 to 0.03 mg/mL.

### (3) Sensitization and Challenge of the Rats

Animals were anesthetized by an intramuscular injection of a mixture of ketamine hydrochloride (0.5 mg/kg of body weight), atropine (0.1 mg/kg of body weight), and xylazine (1 mg/kg of body weight).

Sensitization at day 0 and challenge at day 21 were performed by intratracheal instillation of 150 *μ*L of pollen grains (0.5 mg/rat) and/or PCGs (4.5 × 10^7^/rat) in aqueous suspension used as allergen sources.

For allergic interactive effects, 2 groups were studied. Rats of the "PG" group were sensitized with pollen and challenged with PCGs. Rats of the "GP" group were sensitized with PCGs and challenged with pollen. For a comparison to the previous groups, 2 other groups were also studied. In these groups, sensitization and challenge were performed with the same aeroallergen: "PP" group rats sensitized and challenged with only pollen grains and "GG" group rats sensitized and challenged with only PCGs. A last group served as negative control (NC): animals received only saline solution (Table 1).

**Table 1:** Experimental Design

	Allergen Sources		
	
Group	n*	Sensitization--Day 0	Challenge--Day 21
Negative control	6	NaCl 0.9%	
PP	6	Pollen grains	
GG	6	Isolated PCG	
PG	6	Pollen grains	Isolated PCG
GP	6	Isolated PCG	Pollen grains

*Number of rats per group; total rats studied, 30.

### (4) Autopsy

At day 25, rats were anesthetized by intraperitoneal injection of pentobarbital (150 mg/kg of body weight). They were exsanguinated by cutting the inferior vena cava after blood was taken. Bronchoalveolar lavage fluids, blood samples, and bronchial lymph nodes were collected.

### (5) Serum Sample

After collection, blood was kept at 4°C for about 2 to 4 hours for clotting. The blood samples were then centrifuged for 10 minutes at 2000 *g *at 4°C. Serum was removed and stored in 500-*μ*L aliquots at -80°C until use.

### (6) Bronchoalveolar Lavage Fluid

Lungs were washed 3 times with 10 mL of phosphate-buffered saline (pH 7.2). Fluid collected from the bronchoalveolar lavage of each animal was centrifuged at 150 *g *for 10 minutes.

Different parameters were studied in bronchoalveolar lavage fluid (BALF), as outlined below.

#### (a) BALF alveolar cells

BALF alveolar cells were counted and then isolated by centrifugation (300 rpm, 5 min). Cell differential counts were performed after May-Grünwald Giemsa cytospin slide preparation.

Cell-free BALF was concentrated using Amicon Ultra tubes (Millipore) until the volume was equal to 1 mL and then protein concentrations were determined by the Lowry method [15].

#### (b) Cytokines

Cytokines were quantified in concentrated BALF, first, using a Bio-Plex kit for IL-1*α*, IL-1*β*, IL-2, IL-4, IL-6, IL-10, interferon-*γ *(IFN*γ*), and tumor necrosis factor *α *(TNF*α*) (catalog no: 171K11070, Bio-Rad, France) and, second, rat cytokine multiplex immunoassay kit for IL-5, IL-13, and Rantes (catalog no. RCYTO-80k-04, Linko, Millipore, France) according to the manufacturer's instructions.

IL-4, IL-5, and IL-13 were considered as T_H_2 cytokines and IFN*γ *was taken as the T_H_1 prototypic cytokine.

The T_H_2/T_H_1 ratio, allowing a more synthetic appreciation between T_H_2 and T_H_1 cytokine levels, was calculated as follows: Th2/Th1 ratio = (IL-4 + IL-5 + IL-13)/IFN*γ*.

### (7) Enzyme-Linked Immunosorbent Assay for Specific Anti-Timothy Pollen IgE and IgG1

The enzyme-linked immunosorbent assay was performed in serum according to previous studies by our team [9,10,16].

### (8) Bronchial Lymph Node Cell Assay

The bronchial lymph node assay previously described [9,10] has been slightly modified. Briefly, isolated lymph nodes cells were incubated, with and without allergen sources (pollen, 100 *μ*g/mL; PCGs, 9 × 10^6 ^PCGs/mL), for 3 days in a humidified atmosphere. Then, cells were incubated for 24 hours with 10 *μ*L of [^3^H]-thymidine (Amersham, Bucking-hamshire, UK, 37 kBq/mL) per well and harvested for scintillation counting in a *β*-plate counter (1205 Betaplate Wallac, Turku, Finland) to measure cell proliferation. T-cell reactivity was expressed as counts per minute (cpm).

### (9) Statistical Analysis

The results of all studied parameters are expressed as mean ± SEM. For statistical analysis, the nonparametric tests (Kruskall-Wallis and Mann-Whitney) were used. The results were considered significant when a *P *value less than 0.05 was found.

## Results

### (1) Pollen-Specific IgE and IgG1 Levels in Rat Sera

In contrast to the NC group, the PP rats' group presented a high increase of IgE and IgG1 levels in sera and the GG group presented a low increase for these 2 immunoglobulins. In both cases, these increases were statistically significant.

**Figure 1:**
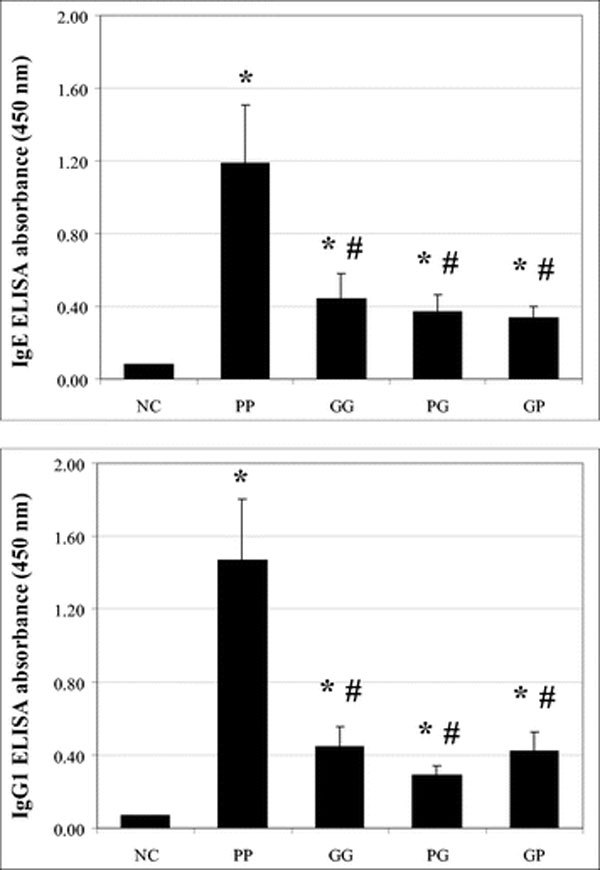
**Timothy grass pollen-specific IgE and IgG1 levels in sera (mean ± SEM)**. NC, negative control group; PP, rats sensitized and challenged with 0.5 mg of pollen; GG, rats sensitized and challenged with 4.5 × 10^7 ^PCGs; PG, rats sensitized with 0.5 mg of pollen and challenged with 4.5 × 10^7 ^PCGs; GP, rats sensitized with 4.5 × 10^7 ^PCGs and challenged with 0.5 mg of pollen; *, significantly different from the NC group (*P *< 0.05); **#**, significantly different from the PP group (*P *< 0.05).

Compared with the NC group, the PG and GP rats' group had significantly high IgE and IgG1 levels. These 2 groups presented significantly lower IgE and IgG1 levels than the PP group, but no significant difference in IgE and IgG1 levels with the GG group (Figure 1).

### (2) Protein Concentrations in BALF

In contrast to the NC group, PP and GG groups presented significant increases of protein concentrations in BALF.

Interactive effects' groups (PG and GP groups) presented significantly higher protein concentrations in comparison to the NC group. The protein concentrations of these rats' groups were higher than those obtained for PP and GG groups, but not statistically significant (Table 2).

**Table 2:** Protein Concentrations and Cytokine Levels (Mean ± SEM) in BALF

	NC	PP	GG	PG	GP
Protein (mg/mL)	0.66 ± 0.09	1.79 ± 0.19*	1.85 ± 0.27*	2.23 ± 0.18*	2.44 ± 0.30*
Pro-inflammatory cytokines (pg/mL)					
IL-2	1.3 ± 0.7	2.8 ± 1.0	2.2 ± 0.6	2.4 ± 0.7	4.4 ± 1.3
IL-6	21 ± 8	98 ± 20*	55 ± 18	50 ± 10	74 ± 21
IL-10	4.4 ± 2.7	17.0 ± 3.8*	18.6 ± 4.9*	17.7 ± 3.3*	18.4 ± 6.3*
IFNγ	6.9 ± 5.7	41.8 ± 11.9*	16.6 ± 6.0	18.2 ± 4.7	24.2 ± 9.4
TNF*α*	12 ± 8	71 ± 11*	72 ± 11*	69 ± 18*	86 ± 20*
IL-1*α*	4.8 ± 1.9	8.4 ± 1.8	9.9 ± 1.5	19.9 ± 1.7*†‡	27.7 ± 6.1*†‡
IL-1*β*	120 ± 20	186 ± 31	257 ± 40*	627 ± 62*†‡	645 ± 114*†‡
Pro-allergy cytokines (pg/mL)					
IL-4	11.3 ± 7.3	18.4 ± 7.2	22.5 ± 9.6	17.7 ± 8.7	(28.9)§
IL-5	13 ± 6	142 ± 51	316 ± 110*	131 ± 31*	655 ± 226*
IL-13	14.5 ± 6.0	43 ± 9.6	61.0 ± 21.4*	32.7 ± 6.4	51.3 ± 14.7*
Rantes	6.8 ± 1.8	9.9 ± 2.2	22.4 ± 3.1*†	26.1 ± 2.2*†	31.4 ± 4.8*†
Th2/Th1 ratio¶	5.62 ± 4.84	4.86 ± 0.3	24.06 ± 0.2*†	9.96 ± 0.1‡	30.4 ± 2.2*†

*Significantly different to NC (*P *< 0.05).

**†**Significantly different to PP group (*P *< 0.05).

‡Significantly different to GG group (*P *< 0.05).

§ In this group, 1 rat only presented IL-4 in BALF.

¶Th2/Th1 ratio = (IL-4 + IL-5 + IL-13)/(IFNγ).

NC, negative control group; PP, rats sensitized and challenged with 0.5 mg of pollen; GG, rats sensitized and challenged with 4.5 × 10^7 ^PCG; PG, rats sensitized with 0.5 mg of pollen and challenged with 4.5 × 10^7 ^PCG; GP, rats sensitized with 4.5 × 10^7 ^PCG and challenged with 0.5 mg of pollen.

### (3) Cytokines in BALF

#### (a) Pro-inflammatory cytokines (Table 2)

In contrast to the NC group, the PP group presented significant increases of IL-6, IL-10, IFNγ, and TNF*α *levels in BALF. The GG group presented significant increases of IL-1*β*, IL-10, and TNF*α *compared with the NC group.

Interactive effects' groups (PG and GP groups) presented significant increases of IL-1*α*, IL-1*β*, IL-10, and TNF*α *levels compared with the NC group. Although these interactive groups had lower IL-6 and IFN*γ *levels in BALF than the PP group, no statistically significant difference was shown. PG and GP groups had significantly higher IL-1*α *and IL-1*β *levels than the GG group.

#### (b) Pro-allergy cytokines (Table 2)

In contrast to the NC group, the GG group presented significant increases of IL-5, IL-13, and Rantes levels. The NC and PP groups presented no significant differences for these cytokine levels.

PG rats' group presented significant increases of IL-5 and Rantes levels than the NC group. The PG group presented a significant increase of Rantes levels in BALF compared with the PP group. Although the PG group presented lower IL-5 and IL-13 levels than the GG group, no statistically significant difference was shown.

Compared with the NC group, the GP group presented significant increases of all pro-allergy cytokines' levels. Rats sensitized with PCGs and challenged with pollen grains (GP group) presented higher IL-5, IL-13, and Rantes levels than the PP group, but the difference was statistically significant only for the Rantes level. The GP rats' group showed higher IL-5 levels than those obtained for the GG group, but this increase was not statistically significant.

#### (c) T_H_2/T_H_1 ratio (Table 2)

In contrast to the NC group, the PP group presented lower values of the T_H_2/T_H_1 ratio, but they were not statistically significant. However, these ratio values were significantly higher for the GG group than for the NC group.

Rats sensitized with pollen grains and challenged with PCGs (PG group) presented no significant difference in T_H_2/T_H_1 ratio in comparison with NC and PP groups. The PG group had significantly lower values of this ratio in comparison with the GG group.

The GP group presented significantly higher values of the ratio than those obtained in NC and PP groups. GP showed also a higher T_H_2/T_H_1 ratio than the GG group, but this difference was not statistically significant.

### (4) Alveolar Cells in BALF

PP and GG groups presented a significantly higher macrophage, eosinophil, and neutrophil numbers in BALF than the NC group. The BALF lymphocyte numbers were significantly higher for GG rats' group than for the NC group.

**Figure 2:**
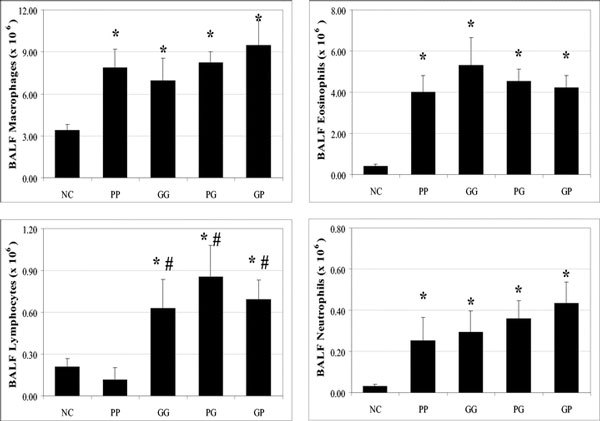
**Alveolar cells (mean ± SEM) in BALF**. Macrophages, eosinophils, lymphocytes, and neutrophils were counted. NC, negative control group; PP, rats sensitized and challenged with 0.5 mg of pollen; GG, rats sensitized and challenged with 4.5 × 10^7 ^PCGs; PG, rats sensitized with 0.5 mg of pollen and challenged with 4.5 × 10^7 ^PCGs; GP, rats sensitized with 4.5 × 10^7 ^PCGs and challenged with 0.5 mg of pollen; *, significantly different from the NC group (*P *< 0.05); **#**, significantly different from the PP group (*P *< 0.05).

Compared with the NC group, PG and GP groups had statistically significant high numbers for all alveolar cells in BALF. Interactive effects' groups (PG and GP) presented significantly higher lymphocyte numbers than the PP group. PG and GP groups presented no significant difference for all alveolar cell numbers in BALF compared with the GG rats' group (Figure 2).

### (5) Lymph Node Cell Proliferation Induced by Pollen or PCGs

After in vitro addition of pollen grains (100 *μ*g/mL) or PCGs (9 × 10^6 ^PCGs/mL), rats sensitized and challenged with the same aeroallergen (pollen grains or PCGs) presented a significantly higher lymph node cell proliferation than the NC group.

In both cases, rats sensitized with pollen grains and challenged with PCGs (PG group) presented significantly higher proliferation than the NC group. The proliferative responses of the PG rats' group presented no significant difference than those obtained with lymph node cells of PP and GG groups.

**Figure 3:**
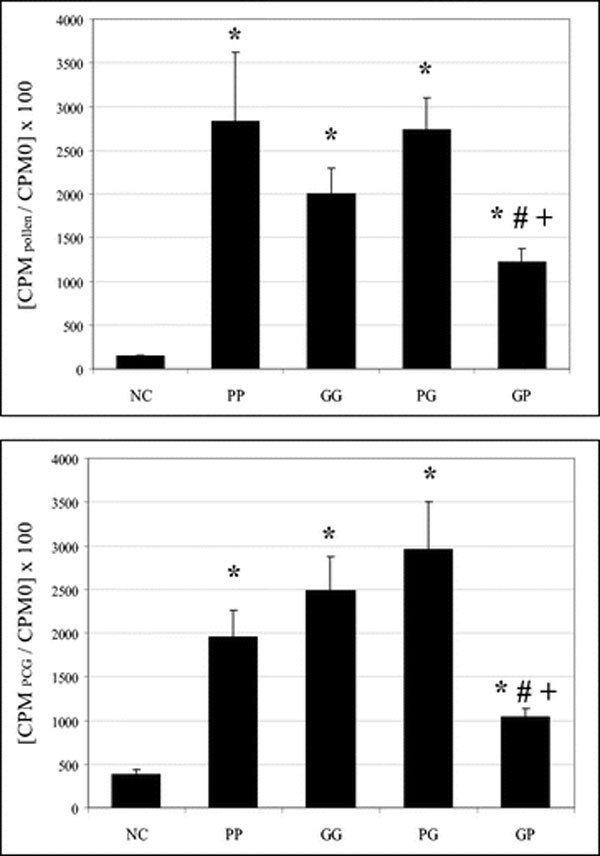
**Pollen and PCGs-induced proliferation of lymph node cells**. Cells from the NC and the 4 sensitized rats' groups were cultured with [^3^H]-thymidine in the presence of 100 *μ*g of pollen and 9 × 10^6 ^PCGs. cpm_pollen_, thymidine incorporation by pollen-stimulated cells; cpm_PCGs_, thymidine incorporation by PCGs-stimulated cells; cpm_0_, thymidine in-corporation in control cells; NC, negative control group; PP, rats sensitized and challenged with 0.5 mg of pollen; GG, rats sensitized and challenged with 4.5 ×10^7 ^PCGs; PG, rats sensitized with 0.5 mg of pollen and challenged with 4.5 × 10^7 ^PCGs; GP, rats sensitized with 4.5 × 10^7 ^PCGs and challenged with 0.5 mg of pollen; *, significantly different from the NC group (*P *< 0.05); **#**, significantly different from the PP group (*P *< 0.05); +, significantly different from the GG group (*P *< 0.05).

The GP group, after in vitro addition of pollen grains pollen or PCGs, presented significantly higher lymph node cell proliferation than the NC group. The GP rats' group showed significantly lower lymph node cell proliferation than the PP and GG groups (Figure 3).

## Discussion

In this study, immunologic interactive effects between timothy grass pollen grains and their PCGs were performed by an evaluation of allergy (humoral and cellular responses) and inflammatory parameters on Brown Norway rats, a good animal model of pollen allergy [17]. In parallel, allergic and inflammatory responses of these interactive effects were compared with those obtained on rats sensitized and challenged with only one aeroallergen, pollen grains or PCGs.

Our findings showed that combined effects between pollen and PCGs induce humoral responses. Previous studies showed that PCGs and pollen grains induced humoral responses on Brown Norway rats [9,10,16] when each aeroallergenic source was used for both sensitization and challenge. PCGs can also trigger IgE-mediated reactions in grass pollen-sensitive patients [4].

Humoral responses on allergic PG and GP groups were influenced mainly by PCGs. On these groups (PG and GP groups), the IgE and IgG1 levels were not different from those obtained for GG rats' group, but they were lower than those obtained for the PP group. The difference of the quantity and quality of allergens present on isolated PCGs versus whole pollen grains could explain the difference between allergic interactive effects groups (PG and GP) and pollen group (PP). Indeed, a solution of whole pollen contains water-soluble and water-insoluble allergens from pollen grains, free-cytoplasmic pollen allergens, and water-soluble and water-insoluble allergens from PCGs [18,19]. A PCGs solution contains mainly water-insoluble allergens from PCGs with few water-soluble allergens of pollen grains and PCGs. Therefore, a loss of water-soluble allergens could arise from PCGs washing steps (data not shown). We can also consider the fact that, after one sensitization and a single challenge, humoral responses on interactive effects' groups seem to be influenced by the aeroallergen that induced the lowest responses. In this study, the aeroallergenic source inducing the lowest responses was PCGs.

As humoral responses, interactive effects between PCGs and pollen induced cellular responses, but these responses varied according to the different parameters studied.

For all immunized animals on pollen grains and/or PCGs, a higher proliferation of lymph node cells was observed when compared with those of the NC group. In the present study, the T-mediated cell responses were dissimilar: the animals sensitized to pollen grains and challenged by PCGs (PG group) have globally the same profile as the rats immunized only either to pollen or to PCGs (PP and GG groups). In contrast, with the inverse protocol (meaning first sensitization by PCGs and second step challenge by pollen grains, the GP group), a less important lymph node cell proliferation was obtained than those found with only PP or GG groups. In vitro, after addition of PCGs or pollen grains, our team already showed a similar proliferation of lymph node cells of PP and GG rats' groups [9,10,16].

Our study reveals that both interactive effects induce an increase of eosinophils and lymphocytes in BALF in contrast to the NC group.

BALF eosinophil levels were similar for all immunized rats. Our results are similar on this point to those found in other previous studies. Mice sensitized with pollen and challenged with PCGs presented an accumulation of eosinophils in the peribronchial area [20]. Furthermore, Wark et al [21] showed that patients with thunderstorm-related asthma, for whom asthma is suspected to be caused by PCGs, presented higher sputum eosinophils than those with non-thunderstorm-related asthma. However, in our study, sensitization and/or challenge with PCGs (GG, PG, and GP groups) induce in BN rats greater lymphocyte levels in BALF than for those of the pollen group. In humans, only patients with severe asthma and asthma-related deaths presented lymphocyte infiltrations in the bronchial walls [22].

In this study, among pro-allergy cytokines, IL-4 levels in sensitized rats were not significantly different from what has been obtained in the NC group. In contrast to IL-4, the sensitization with PCGs (GG and GP groups) induced an increase of IL-5 and IL-13 levels in BALF when compared with the NC group, but rats sensitized with pollen (PP and PG groups) presented no significant difference in IL-5 and IL-13 levels with the NC group. Whatever sensitization and/or challenge was used (GG, PG, and GP groups), PCGs increased Rantes levels in BALF. In patients with allergies, some studies showed that, in BALF, IL-5 and IL-13, but not IL-4, levels increased when compared with those of healthy patients [23-25]. People with asthma also presented an increase of Rantes level in BALF during the pollen season compared with their Rantes level measured before pollen season [26]. Furthermore, patients with asthma but not patients with seasonal allergic rhinitis have increased levels of Rantes in sputum compared with healthy subjects [27].

The increase of T_H_2/T_H_1 ratio and the IL-5 and IL-13 levels in the BALF of rats that were sensitized with PCGs (GG and GP groups) suggest that the sensitization step was an important factor in the allergic reaction. As a matter of fact, the increase in Rantes and lymphocytes on rats sensitized and/or challenged with PCGs (GG, PG, and GP groups) suggests that interactive effects' groups seem to be influenced mainly by PCGs. Furthermore, PCGs could induce asthma when, in contrast, whole pollen is more implicated in the triggering of hay fever. This hypothesis is supported by other studies on patients with allergies/asthma [4,28].

Interactive effects between pollen grains and PCGs induced inflammation in Brown Norway rats. Compared with the pollen and PCGs group, PG and GP groups presented identical macrophage and neutrophil numbers in BALF. In this work, other inflammatory parameters, such as protein concentrations and pro-inflammatory cytokine levels in BALF, led to the belief that interactive effects between pollen grains and PCGs induced a stronger inflammation than those obtained on PP and GG groups. The presence of NAD(P)H oxidase [20,29] and (1 → 3)-*β*-glucan,[30] in pollen grains and PCGs, could partly explain airways inflammation.

In conclusion, different elements of our study give several tracks for a better understanding of the immunologic interactive effects between pollen grains and PCGs issued from *Phleum pratense*. Even if it is currently only based on timothy grass pollen grains, this new approach combining, for the first time, pollen and PCGs for immunization shows that multiple joint contacts with pollen and PCGs can lead, at least with BN rats, to specific IgE responses and, more globally, inflammatory phenomena. For humoral and cellular allergic responses, immunologic interactive effects between the 2 aeroallergenic sources used in this study seem to be influenced mainly by PCGs. But interactive effects seem to induce a stronger inflammation than pollen or PCGs when they are used for both sensitization and challenge.
